# Multi-Sensor Passive Localization Using Direct Position Determination with Time-Varying Delay

**DOI:** 10.3390/s19071541

**Published:** 2019-03-29

**Authors:** Shangyu Zhang, Zhen Huang, Xuefeng Feng, Jiazhi He, Lei Shi

**Affiliations:** 1 School of Aerospace Engineering, Tsinghua University, Beijing 100084, China; zhangshangyu13@mails.tsinghua.edu.cn (S.Z.); fxf17@mails.tsinghua.edu.cn (X.F.); hjz15@mails.tsinghua.edu.cn (J.H.); shilei16@mails.tsinghua.edu.cn (L.S.); 2 Beijing National Research Center for Information Science and Technology, Tsinghua University, Beijing 100084, China; 3 Space Center, Tsinghua University, Beijing 100084, China

**Keywords:** passive localization, direct position determination, time-varying delay, maximum likelihood

## Abstract

This paper focuses on passive emitter localization using moving sensors. The increase in observation time is beneficial to improve the localization accuracy, but it could cause deterioration of the relative motion between the emitter and the sensors, especially the nonlinear motion. The common localization algorithms typically have two steps: (1) parameter estimation and (2) position determination, where the parameters are assumed to be constant, and it is not applicable for long observation times. We proposed the time-varying delay-based direct position determination (DPD-TVD) method, regarding the variation in the propagation time delay during the observation time. Using one step, the proposed algorithm can obtain the emitter’s position directly from the received signals by calculating the cost function corresponding to the map grid. By better adapting to highly dynamic scenarios, the proposed algorithm can achieve better localization accuracy than that of constant parameters using one-step or two-step procedures, which is demonstrated by the simulation results.

## 1. Introduction

Passive localization of electronic emitters has always been an important topic in disciplines such as signal processing, communications, and acoustics. Conventional algorithms for determining the position of noncooperative emitters are based on the initial estimation of certain parameters, such as angle of arrival (AOA) [1], received signal strength (RSS) [2], time of arrival (TOA) [3,4], time difference of arrival (TDOA) [5], frequency difference of arrival (FDOA) [6,7] or rate of frequency difference of arrival (RFDOA) [8]. These intermediate parameters are then used for the localization of the emitter based on the known positions and velocities of the sensors. Such two-step localization algorithms are sub-optimal from the point of view of estimation, as some loss of information during data processing is inevitable.

Weiss [9] proposed the Direct Position Determination (DPD) approach, which obtains the emitter position directly from the received signals. Combining observations from all sensors, the DPD algorithm can estimate the emitter’s location by constructing a simple closed-form maximum-likelihood (ML) cost function that depends only on the location and search space of geographic grids. Amar and Weiss [10] proposed a DPD algorithm based on the Doppler frequency shift for narrowband signals, and Weiss [11,12] developed a ML location DPD algorithm for wideband random signals using both the Doppler effect and the relative delay. Li [13] used a coherent summation method that takes the coherency among the short-time signals received at the same receiver into account, while Pourhomayoun [14,15] introduced a complex ambiguity function (CAF)-based algorithm to find a balance between localization performance and computation. In [16], Vankayalapati used a continuous time model and derived a TDOA-based DPD estimator. Steffes [17] proposed a DPD algorithm for single-sensor TDOA localization, and Tzoreff [18] developed an expectation-maximization-based DPD algorithm to replace the high-dimensional search process with several one-dimensional searches. Lu [19] used a single iterative particle filter algorithm to overcome the problems associated with high computational loads. The DPD algorithms mentioned above [10,11,12,13,14,15,16,17,18,19] use sensors with a single antenna. Some studies [9] focused on sensors equipped with array antennas that can utilize AOA information. Tirer and Tzafri proposed high-resolution DPD algorithms [20,21], and DPD algorithms have been developed for special signals such as cyclostationary [22] and strictly noncircular signals [23]. The influence of model error on the localization accuracy was analyzed in [24,25]. DPD algorithms have also been extended to the case of multiple emitters [26,27,28,29].

Both the one-step and two-step algorithms use the geometry and relative motion between emitter and sensors. If the signal models are assumed to have the same order, the Cramer–Rao lower bound (CRLB) that can be achieved by the one-step and two-step methods is asymptotically the same [11]. The increase in observation time is beneficial as it improves the localization accuracy. However, as the traditional models of one-step or two-step algorithms assume parameters to be constant, the observation time cannot be increased beyond a certain limit, because it could cause deterioration as the relative motion between the emitter and the sensors, especially the nonlinear motion. Hu [30] introduced the relative time companding (RTC) and relative Doppler companding (RDC) factors to analyze the problem quantitatively. If the observation time exceeds the limitation, the shape of cost function used for parameter estimation in two-step algorithms will expand, resulting in performance degradation and possibly the failure of localization. The same problem occurs in the DPD algorithms, which are investigated in this paper. If the observation time is large, the cost function shape that corresponds to the map grid will expand. Therefore, the traditional signal model based on time delay and Doppler shift [11] is no longer suitable for localization with long observation time. To prevent the cost function shape of CAF from expanding, Hu [8] estimated RFDOA along with TDOA and FDOA using three steps. In addition, the estimated RFDOA can be used for localization, which is conducive to enhancing the accuracy. However, there is no DPD algorithm that considers the long observation time problem in highly dynamic scenarios.

In this paper, we propose a DPD algorithm that uses a time-varying time delay for passive localization with long observation time in highly dynamic scenario. We regard the time delay as a time-varying parameter during the observation time, where the time delay of each sampling point is calculated. The time-varying delay model contains all the information about the delay and relative motion between the sensors and the emitter. In the case of short observation times, constant models of traditional methods are special cases of the time-varying delay model. This paper derives the CRLB of the proposed algorithm and demonstrates the algorithm’s performance using simulations.

The remainder of this paper is organized as follows. The signal model and problem formulation are introduced in Section 2. In Section 3, the DPD-TVD algorithm is proposed. Experimental and simulation results are presented in Section 5 to validate our algorithm, and we also illustrate the performance through a comparison with the traditional DPD and CRLB. Finally, the conclusions are summarized in Section 7.

## 2. Signal Model and Problem Formulation

The transmitted signal model is given by

(1) $$s\left(t\right)=a\left(t\right)e^{j2\pi f_{c}t},$$

where $f_{c}$ is the carrier frequency, a(t) is the signal envelope. −T/2≤t≤T/2, and *T* is the observation time. Note that the signal bandwidth *B* of a(t) satisfies $B\;\ll \;f_{c}$.

**Figure 1:**
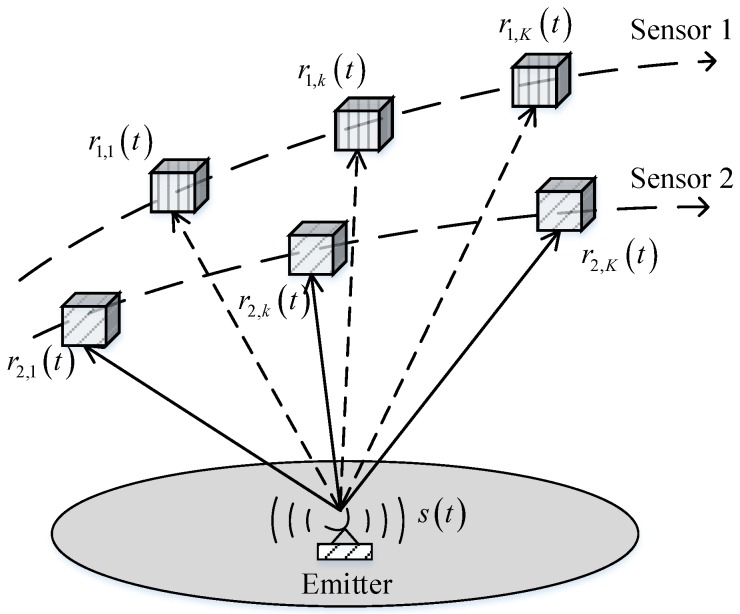
The localization scenario.

As shown in Figure 1, consider a stationary radio emitter located at p and *L* moving sensors whose frequencies and times are synchronized. The sensors are on platforms moving in the air, such as satellites, aircrafts or UAVs, etc. and use navigation device such as GPS to obtain their position and velocity. The *L* sensors intercept the transmitted signals in *K* short intervals. The signal observed by sensor *l* in interception interval *k* is given by

(2) $$r_{l,k}\left(t\right)=b_{l,k}a_{k}\left(t-\tau_{l,k}\left(t\right)\right)e^{j2\pi f_{c}\left(t-\tau_{l,k}\left(t\right)\right)}+\omega_{l,k}\left(t\right),$$

where $a_{k}\left(t\right)$ is the signal envelope at interception interval *k*, $\tau_{l,k}\left(t\right)$ is the time-varying delay, $b_{l,k}$ is the channel transmission attenuation, and $\omega_{l,k}\left(t\right)$ is white Gaussian noise. In contrast to the traditional algorithms, we consider the propagation time as a time-varying variable during each interception interval. This hypothesis is applicable to highly dynamic scenarios in which the emitter and sensors exhibit nonlinear relative motion.

We define the time-varying delay as

(3) $$\tau_{l,k}\left(t\right)≜\frac{1}{c}∥p-p_{l,k}\left(t\right)∥,$$

where $p_{l,k}\left(t\right)$ denotes the coordinate vector of sensor *l* at interception interval *k*.

To compare the time-varying signal model with the traditional signal model, the time-varying variable τ(t) is expressed as a Taylor series at the point when *t* = 0 by

(4) $$\tau \left(t\right)=\tau \left(0\right)+\tau^{\prime }\left(0\right)t+\frac{1}{2}\tau^{\prime \prime }\left(0\right)t^{2}+\cdots ,$$

where τ(0), $\tau^{\prime }\left(0\right)$, $\tau^{\prime \prime }\left(0\right)$, and $\tau^{\prime \prime \prime }\left(0\right)$ are defined as

(5) $$\begin{array}{cccc} \tau (0) & =\frac{r}{c}=t_{r},\; & \tau^{\prime }\left(0\right) & =\frac{\dot{r}}{c}=\frac{f_{r}}{f_{c}},\\ \tau^{\prime \prime }\left(0\right) & =\frac{\ddot{r}}{c}=\frac{{\dot{f}}_{r}}{f_{c}}, & \tau^{\prime \prime \prime }\left(0\right) & =\frac{\dddot{r}}{c}=\frac{{\ddot{f}}_{r}}{f_{c}}. \end{array}$$

In (5), *r*, $\dot{r}$, $\ddot{r}$, and $\dddot{r}$ are the propagation distance, relative velocity, relative acceleration, and acceleration jerk between the emitter and sensor, respectively, when *t* = 0. $t_{r}$, $f_{r}$, ${\dot{f}}_{r}$, and ${\ddot{f}}_{r}$ are the time delay, Doppler shift, Doppler shift rate, and rate of Doppler shift rate, respectively.

According to [30], the relative Doppler companding factor is defined as

(6) $$\gamma_{1}=\frac{\ddot{r}T^{2}}{\lambda }={\dot{f}}_{r}T^{2},$$

where $\lambda =c/f_{c}$ is the signal wavelength. In (6), $\gamma_{1}$ is the ratio of the Doppler compand ${\dot{f}}_{r}T$ with respect to the FDOA resolution 1/T. For example, $\gamma_{1}$ = 2 means that the Doppler compand is two times as much as the FDOA resolution. As mentioned in [30], if $\gamma_{1}>4$, the time delay and Doppler shift-based signal model will be invalid and higher order parameters have to be considered.

Similarly, a normalized factor for the time delay, Doppler shift, and the Doppler shift rate-based model can be defined as the relative Doppler Rate companding factor. As shown by the result in [30], the RFDOA resolution is $1/T^{2}$. The Doppler rate compand is ${\ddot{f}}_{r}T$. So, the factor can be given by

(7) $$\gamma_{2}=\frac{\dddot{r}T^{3}}{\lambda }={\ddot{f}}_{r}T^{3}.$$

In certain scenarios, (6) and (7) can be used to calculate the thresholds of the observation time when the time delay and Doppler shift-based signal model and the time delay, Doppler shift, and Doppler shift rate-based signal model become invalid.The thresholds are given by

(8) $$\begin{array}{cc} T_{\gamma_{1}} & =\sqrt{4\lambda /\ddot{r}}\\ T_{\gamma_{2}} & =\sqrt[{3}]{4\lambda /\dddot{r}}. \end{array}$$

Substituting the first two terms of Taylor series of τ(t) in (4) into the signal model in (2), we obtain

(9) $$\begin{array}{cc} r(t) & =ba\left(t-\tau \left(0\right)-\tau^{\prime }\left(0\right)t\right)e^{j2\pi f_{c}\left(t-\tau \left(0\right)-\tau^{\prime }\left(0\right)t\right)}\\ & ≅ba\left(t-t_{r}\right)e^{j2\pi f_{c}\left(t-t_{r}\right)}e^{-j2\pi f_{r}t}\\ & =bs\left(t-t_{r}\right)e^{-j2\pi f_{r}t}. \end{array}$$

which is the time delay and Doppler shift signal model used in [11]. The traditional model is a special case of time-varying delay model. As the observation time gets longer, the signal model cannot be approximated by using only two terms of Taylor series. The proposed time-varying delay signal model takes not only the time delay and Doppler shift into account, but also, the Doppler shift rate and its higher-order components. This enables the proposed model to prevent performance deterioration and achieve better performance, especially in highly dynamic scenarios.

We define the vectors in (2) as

(10) $$\begin{array}{cc} s_{k} & ≜{\left[s_{k}\left(t_{1}\right),\ldots ,s_{k}\left(t_{N}\right)\right]}^{T}\\ r_{l,k} & ≜{\left[r_{l,k}\left(t_{1}\right),\ldots ,r_{l,k}\left(t_{N}\right)\right]}^{T}\\ w_{l,k} & ≜{\left[\omega_{l,k}\left(t_{1}\right),\ldots ,\omega_{l,k}\left(t_{N}\right)\right]}^{T}\\ D_{l,k} & ≜diag\{e^{2j\pi f_{c}\tau_{l,k}\left(t_{1}\right)},\ldots ,e^{2j\pi f_{c}\tau_{l,k}\left(t_{N}\right)}\}\\ F_{l,k} & ≜{\left[F_{l,k}\left(t_{1}\right),\ldots ,F_{l,k}\left(t_{N}\right)\right]}^{T}, \end{array}$$

where $F_{l,k}\left(t\right)$ is a down shift operator and $F_{l,k}\left(t\right)$ shifts signal $s_{k}$ by $\left\lfloor \tau_{l,k}\left(t\right)/T_{s}\right\rfloor$ integer samples. $w_{l,k}$ is assumed to be Gaussian, and the covariance is $\sigma^{2}I$.

From (2) and (10), we can derive the relation

(11) $$r_{l,k}=b_{l,k}D_{l,k}F_{l,k}s_{k}+w_{l,k},$$

where $b_{l,k}$ is the channel transmission attenuation.

In short, we can briefly state the problem as finding the emitter position using the observations in (11).

## 3. Proposed Algorithm

In this section, we propose a novel DPD algorithm based on the time-varying delay. As can be seen from the previous section, the matrixes $D_{l,k}$ and $F_{l,k}$ contain time-varying delay information. The main concept of DPD is as follows. A reference position is selected in the area where the emitter is thought to be located. Based on the reference position and the known position and velocity information of the sensors, we can calculate the time-varying delay $\tau_{l,k}\left(t\right)$. When the time-varying delay calculated from the reference position minimizes the cost function corresponding to all received signals, we consider the reference point to be the location of the emitter.

As in [9], the estimator can be given in terms of the least-squares principle as

(12) $$\Phi \left(p\right)=\sum_{k=1}^{K}\sum_{l=1}^{L}{∥r_{l,k}-b_{l,k}D_{l,k}F_{l,k}s_{k}∥}^{2}.$$

To minimize (12), we find

(13) $$\begin{array}{cc} {\hat{b}}_{l,k} & ={\left[{\left(D_{l,k}F_{l,k}s_{k}\right)}^{H}D_{l,k}F_{l,k}s_{k}\right]}^{-1}{\left(D_{l,k}F_{l,k}s_{k}\right)}^{H}r_{l,k}\\ & ={\left(D_{l,k}F_{l,k}s_{k}\right)}^{H}r_{l,k}. \end{array}$$

Without loss of generality, we assume that $∥s_{k}{∥}^{2}=1$.

Substituting (13) into (12), we obtain

(14) $$\Phi \left(p\right)=\sum_{k=1}^{K}\sum_{l=1}^{L}∥r_{l,k}{∥}^{2}-{\left|{\left(D_{l,k}F_{l,k}s_{k}\right)}^{H}r_{l,k}\right|}^{2}.$$

Instead of finding the minimum of Φ(p), we determine the maximum of $\tilde{\Phi }\left(p\right)$, which is defined by

(15) $$\begin{array}{cc} \tilde{\Phi }\left(p\right) & =\sum_{k=1}^{K}\sum_{l=1}^{L}{\left|{\left(D_{l,k}F_{l,k}s_{k}\right)}^{H}r_{l,k}\right|}^{2}\\ & =\sum_{k=1}^{K}s_{k}^{H}Q_{k}s_{k}, \end{array}$$

where the vectors are given by

(16) $$\begin{array}{cc} Q_{k} & ≜V_{k}V_{k}^{H}\\ V_{k} & ≜\left[F_{1,k}^{H}D_{1,k}^{H}r_{1,k},\ldots ,F_{L,k}^{H}D_{L,k}^{H}r_{L,k}\right]. \end{array}$$

Note that $V_{k}$ is an L×N matrix that includes information about all of the target positions. The cost function $\tilde{\Phi }\left(p\right)$ can be used to estimate the position of the emitter when the waveform of signal $s_{k}$ is known. However, in actual applications, the waveform of the signal is unknown.

The cost function can be optimized by maximizing each of the *K* quadratic forms with respect to $s_{k}$ [12]. Thus, $s_{k}$ should be selected as the eigenvector corresponding to the largest eigenvalue of $Q_{k}$, which can be defined as $\lambda_{max}\left\{Q_{k}\right\}$.

As *N* becomes larger, the computational cost will increase. For a matrix A, the nonzero eigenvalues of $AA^{H}$ are the same as those of $A^{H}A$; therefore, we have

(17) $$\lambda_{max}\left\{Q_{k}\right\}=\lambda_{max}\left\{{\bar{Q}}_{k}\right\}=\lambda_{max}\left\{V_{k}^{H}V_{k}\right\},$$

which reduces the computational cost when N≫L.

The new cost function can be written as

(18) $$\Theta \left(p\right)=\sum_{k=1}^{K}\lambda_{max}\left\{{\bar{Q}}_{k}\right\}.$$

The (*i*,*j*)-th element of ${\bar{Q}}_{k}$ is given by

(19) $$\begin{array}{cc} {\bar{Q}}_{k}\left(i,j\right) & =V_{i,k}^{H}V_{j,k}\\ & =r_{i,k}^{H}D_{i,k}F_{i,k}F_{j,k}^{H}D_{j,k}^{H}r_{j,k}\\ & ≅\frac{1}{T_{s}}\int_{0}^{T}r_{i,k}^{*}\left[t+\tau_{i,k}\left(t\right)-\tau_{j,k}\left(t\right)\right]r_{j,k}\left(t\right)e^{j2\pi f_{c}\left[\tau_{i,k}\left(t\right)-\tau_{j,k}\left(t\right)\right]}dt. \end{array}$$

This is the CAF [31] of the time-varying delay. $\tau_{l,k}\left(t\right)$ is the function of the target position p, and the cost function can be calculated using the geographical position $p_{r}$. Using a 2D or 3D grid-search approach, the estimated position of the emitter is given by

(20) $$\hat{p}=\arg\max_{p}\Theta \left(p\right).$$

A possible implementation of our time-varying delay DPD framework is given in Algorithm 1.

**Algorithm 1** Algorithm for DPD-TVD1:Define the area in which the emitter may exist and determine an appropriate location grid $p_{i}$, i=1,⋯,M.2:**for** i=1 **to** *M* **do**3: Set $\Theta (p_{i})=0$4: **for** k=1 **to** *K* **do**5:  **for** l=1 **to** *L* **do**6:   Evaluate $\tau_{l,k}\left(t\right)$7:   Evaluate $D_{l,k}$, $F_{l,k}$8:  **end for**9:  Evaluate ${\bar{Q}}_{k}$ and $V_{k}$ according to (16)10:  Let $\Theta \left(p_{i}\right)=\Theta \left(p_{i}\right)+\lambda_{max}\left\{{\bar{Q}}_{k}\right\}$11: **end for**12:**end for**13:**Output**: Find the grid point for which $\Theta (p_{i})$ is largest. This point is the estimated position of emitter $\hat{p}$.14:**End**


## 4. Cramer–Rao Lower Bound

In this section, we focus on the derivation of the Cramer–Rao lower bound (CRLB) of the proposed algorithm. Inspired by the derivation in [11], we obtain the CRLB of DPD-TVD as follows.

The covariance matrix of (11) is

(21) $$\begin{array}{cc} R_{l,k,i,j} & ≜E\{r_{l,k}r_{i,j}^{H}\}\\ & =D_{l,k}F_{l,k}PF_{i,j}^{H}D_{i,j}^{H}\delta_{l,i}\delta_{k,j}+\sigma^{2}I\delta_{l,i}\delta_{k,j}, \end{array}$$

where we assume that the noise is Gaussian and $P≜E\{s_{k}s_{k}^{H}\}$ is the signal covariance matrix.

Some vectors and matrices are defined as

(22) $$\begin{array}{cc} r_{k} & ≜{\left[r_{1,k}^{T},r_{2,k}^{T},\ldots ,r_{L,k}^{T}\right]}^{T}\\ r & ≜{\left[r_{1}^{T},r_{2}^{T},\ldots ,r_{K}^{T}\right]}^{T}\\ R_{k} & ≜E\{r_{k}r_{k}^{H}\}\\ R & ≜E\{rr^{H}\}\\ B_{k} & ≜{\left[F_{1,k}^{H}D_{1,k}^{H},\ldots ,F_{L,k}^{H}D_{L,k}^{H}\right]}^{H}. \end{array}$$

From (22), we have

(23) $$R_{k}=B_{k}PB_{k}^{H}+\sigma^{2}I.$$

The matrix R is block diagonal with *K* blocks, so the Fisher information matrix can be expressed as

(24) $${\left[J\right]}_{i,j}=\sum_{k=1}^{K}\mathrm{tr}\left\{R_{k}^{-1}\frac{\partial R_{k}}{\partial \psi_{i}}R_{k}^{-1}\frac{\partial R_{k}}{\partial \psi_{j}}\right\}.$$

## 5. Simulations

**Figure 2:**
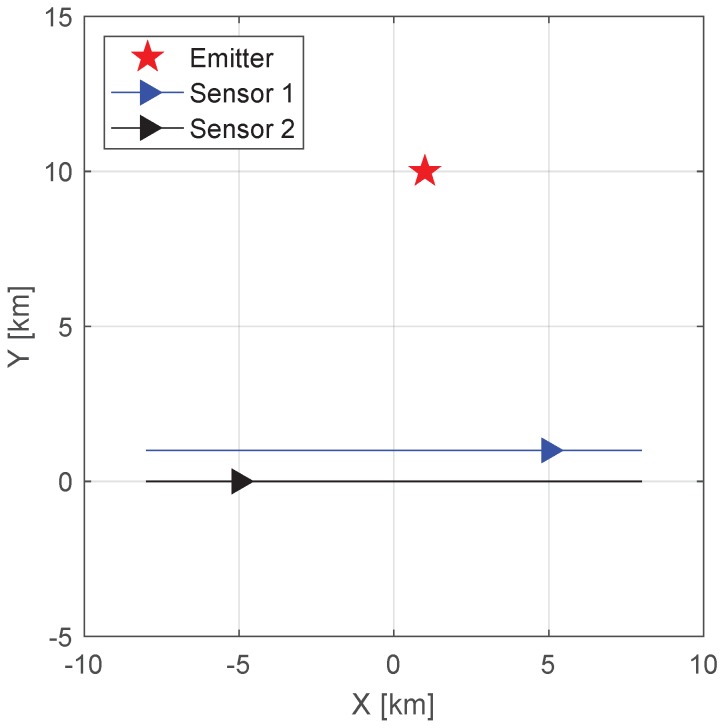
Sensors and emitter geometry. The blue and black triangles represent the positions of sensors 1 and 2 when receiving the signal, the blue and black solid lines represent the trajectories of sensors 1 and 2, and the red pentagram represents the position of the emitter.

This section presents simulation results that demonstrate the localization performance of the proposed algorithm and the CRLB analysis. We consider the 3D scenario depicted in Figure 2. There are two sensors and one emitter. The sensors are located at (5, 1, 20) and (−5, 0, 20) km when intercepting the signal, and the velocities of sensors are (1, 0, 0) and (2, 0, 0) km/s. The stationary emitter is located at (1, 10, 0) km. In this simulation, we have *L* = 2 and *K* = 1. The observation time *T* varies from 0.01 to 1 s in the simulations. The transmitted signal is a binary phase shift keying (BPSK) signal with a bandwidth of *B* = 40 kHz and a carrier frequency of $f_{c}$ = 1 GHz. Throughout the numerical analysis, the signal propagation speed is assumed to be $c=3\times 10^{8}$ m/s. To gather sufficient data, the simulation results are based on 500 Monte-Carlo runs per point.

In the following simulations, we contrast the localization performance of the proposed time-varying delay-based DPD algorithm with the associated CRLB derived in Section 4, denoted by “DPD-TVD” and “CRLB TVD”, respectively. For comparison purposes, the localization results of the TDOA- and FDOA-based two-step algorithm [6], which uses the time delay and Doppler shift signal model, are labeled “2-Step TF”. Additionally, the TDOA-, FDOA-, and RFDOA-based two-step algorithm [8] is also presented, which is labeled “2-step TFRF”. These two signal models are abbreviated as the TF model and TFRF model, respectively.

The DPD algorithm with the time delay and the Doppler shift signal model [11] uses the first- and second-order of the time delay, which is essentially the same as using TDOA and FDOA; it is denoted by “DPD-TF” in the simulations. The CRLBs of the two signal models are denoted by “CRLB TF” and “CRLB TFRF”, respectively.

### 5.1. Cost Function

**Figure 3:**
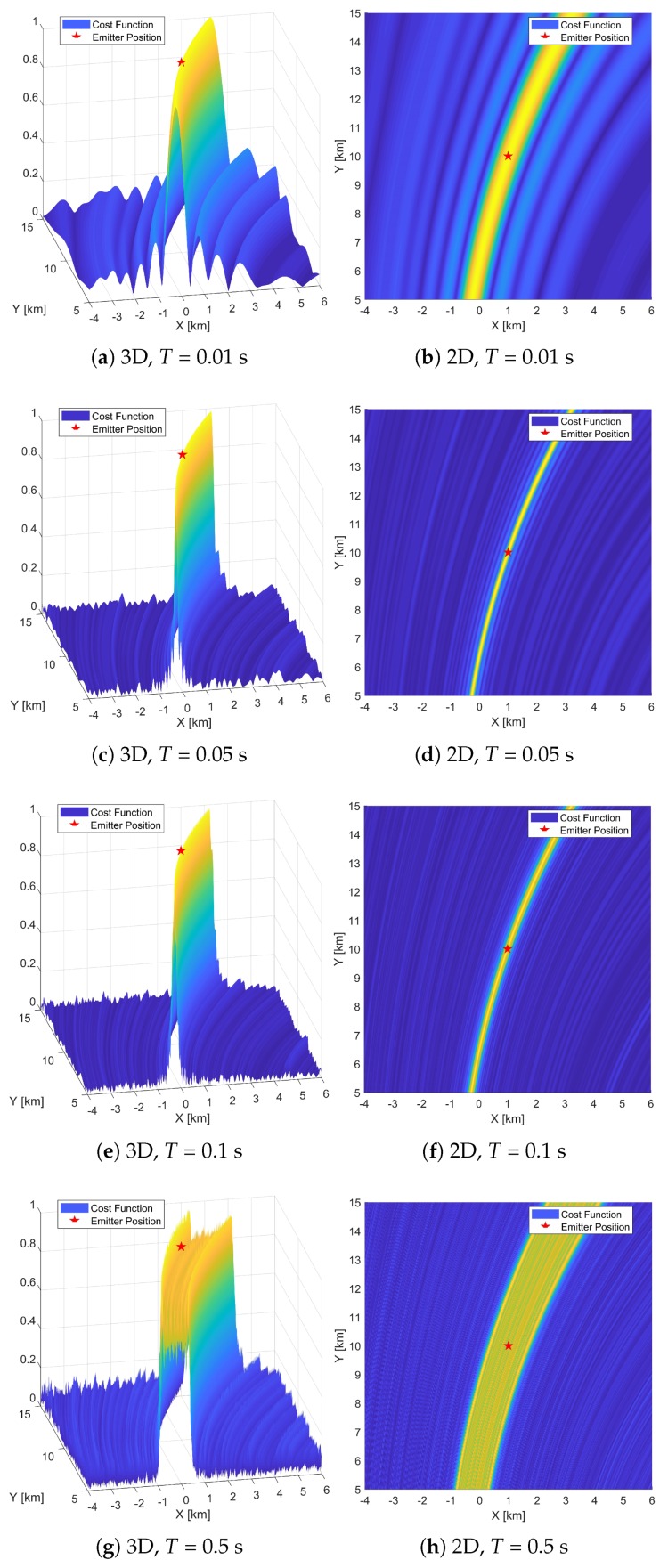
Two-dimensional and three-dimensional cost functions of DPD-TF. *T* = 0.01, 0.05, 0.1, 0.5 s.

**Figure 4:**
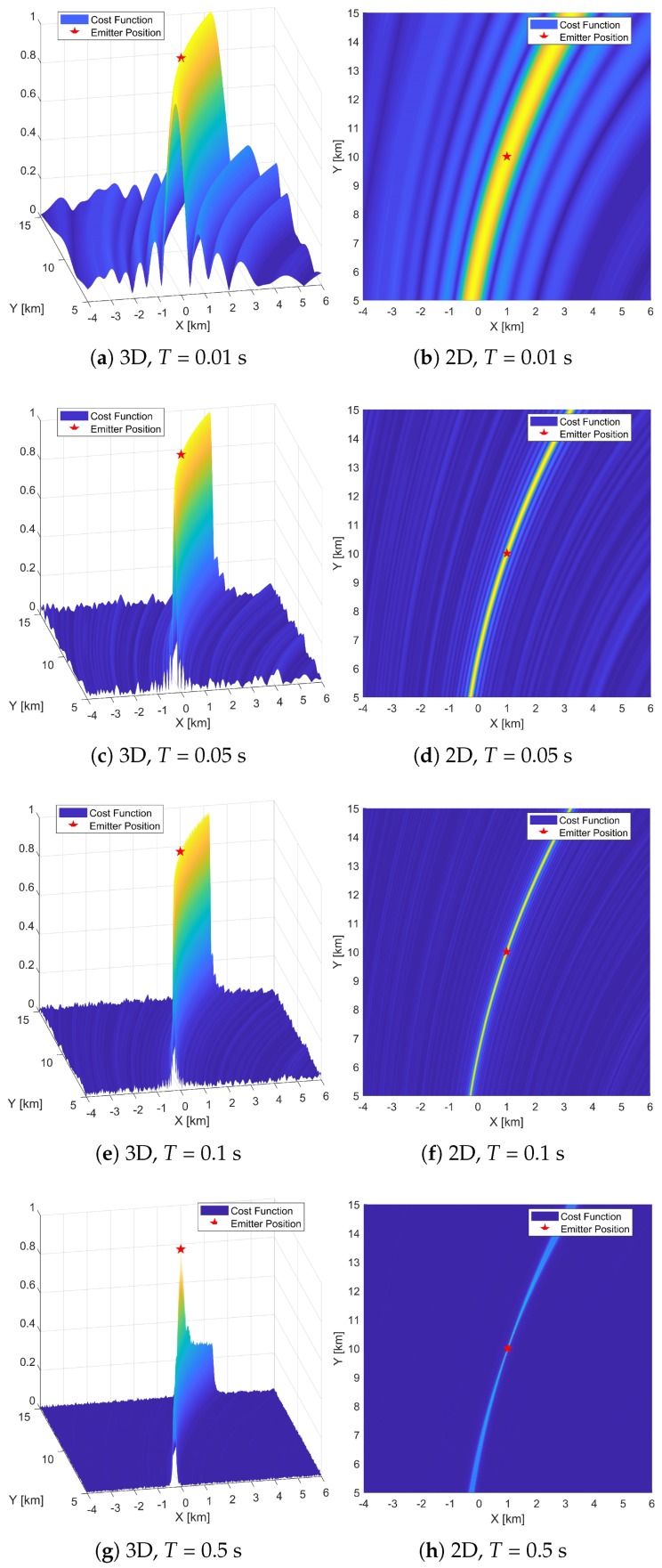
Two-dimensional and three-dimensional cost functions of DPD-TVD. *T* = 0.01, 0.05, 0.1, 0.5 s.

Figure 3 and Figure 4 exhibit the 2D and 3D shapes of cost functions with the DPD-TF algorithm and the proposed DPD-TVD algorithm, respectively. The cost functions are shown at different observation times: *T* = 0.01, 0.05, 0.1, and 0.5 s. Specifically, the left side of Figure 3a–h and Figure 4a–h show the shapes of cost function for 3D plots, and the right side are for 2D plots as a top view. In this simulation, SNR = 10 dB and *B* = 40 kHz. The values are expressed as relative values with a maximum of 1. In all figures, the emitter marked as a red pentagram is located in the exact center of the coverage area. The peak of the cost function should be located at the grid position that is closest to the true emitter position. In the figures, larger values are colored yellow and smaller values are colored blue. According to (8), the thresholds for this scenario are $T_{\gamma_{1}}$ = 0.1 s and $T_{\gamma_{2}}$ = 0.45 s.

By comparing Figure 3a,c, it is apparent that as the observation time increases from 0.01 to 0.05 s, the peak shape becomes sharper. Comparing Figure 3b,d, the width of the yellow portion becomes narrower as *T* increases. As the observation time increases from 0.1 s to 0.5 s, the peak shape broadens slightly along the X-axis. When the observation time reaches 0.5 s, the peak shape broadens from a very narrow peak to one with a width of about 1.2 km along the X-axis, and the edge of the rectangular peak is slightly higher than the vicinity of the center. This indicates that the localization accuracy is prone to degradation as the observation time increases. From Figure 3, we can conclude that by using the time delay and Doppler shift signal model, increasing the observation time in the range $T\leq T_{\gamma_{1}}$ results in a narrower cost function peak shape that is conducive to localization. However, when this range is exceeded, the signal model becomes unsuitable, with the broadening of the cost function signifying a decrease in localization accuracy.

In Figure 4, except for the time-varying delay signal model, the other settings are the same as those used to generate Figure 3. It can be seen from this figure that as the observation time increases from 0.01 to 0.05 s, the cost function peak shape becomes sharper and the width of the peak becomes narrower, which is similar to Figure 3. Comparing Figure 3a–d with Figure 4a–d, we see that the cost function peaks are very similar. This is because for short observation times, the high-order components of the signal have less effect on the cost function. At this point, the two signal models are approximately equivalent. Different from Figure 3, when the observation time exceeds the above range, from 0.05 to 0.1 s, and then to 0.5 s, the peak shape of the cost function becomes increasingly sharp, which is conducive to localization. It can be concluded that the traditional signal model will broaden the peak of the cost function as the observation time exceeds the threshold. The proposed model can guarantee a sharp cost function peak under long observation times.

### 5.2. Distribution of CRLB within the Coverage Area

**Figure 5:**
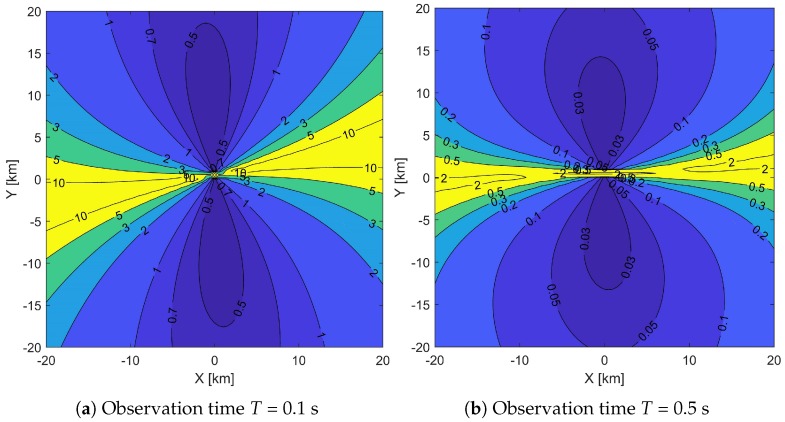
Distribution of Cramer–Rao lower bound (CRLB) within the coverage range with different observation times. Values of accuracy are marked on the contour lines in “km”. Areas with better localization performance are colored blue, and areas with worse performance are colored yellow.

Figure 5 shows the contour distributions of CRLB with the proposed DPD-TVD algorithm within the coverage area. The values of accuracy are marked on the contour lines in “km”. Figure 5a,b exhibit the distributions with observation times of *T* = 0.1 and 0.5 s, respectively. In this simulation, SNR = 10 dB and *B* = 40 kHz. Areas with better localization performance are colored blue, and areas with worse performance are colored yellow. In both subfigures, the accuracy of areas on the top and bottom of the figure are almost symmetric. On the upper and lower sides of the sensors, the localization accuracy is the best. In the areas under the trajectory of sensor and its extension line, the localization accuracy is worst. By quantitatively comparing the accuracy of the two subfigures, the increase in observation time is beneficial to the improvement of localization accuracy.

### 5.3. Dependence on Observation Time *T*

**Figure 6:**
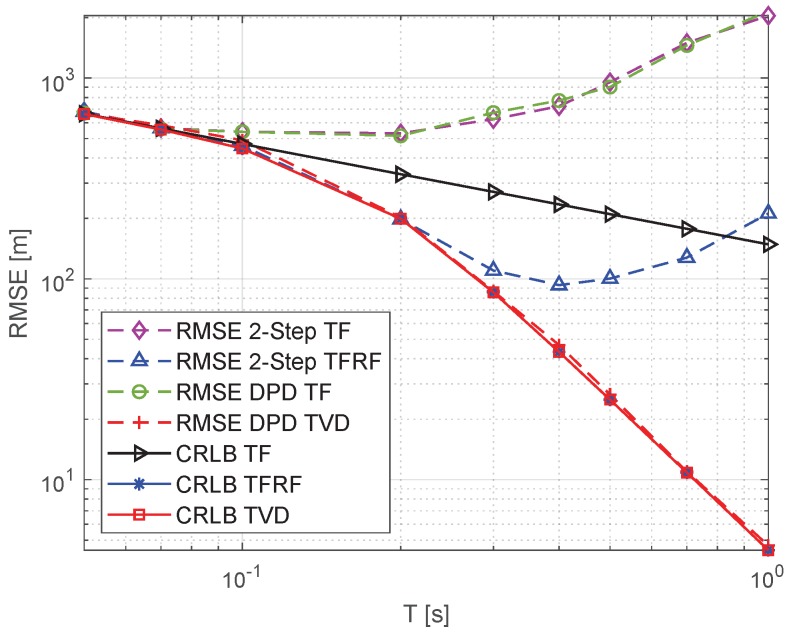
RMSE and CRLB versus *T*. The signal-to-noise ratio (SNR) is 10 dB and signal bandwidth *B* is 40 kHz.

Figure 6 shows the localization accuracy achieved by the different algorithms with respect to the observation time *T*. In these simulations, the signal-to-noise ratio (SNR) is 10 dB and signal bandwidth *B* is 40 kHz. The figure is drawn as a log-log plot, and CRLBs are represented by solid lines, and the root mean squared errors (RMSE) of the algorithm are represented by dashed lines.

When the observation time *T* is less than $T_{\gamma_{1}}$ = 0.1 s, the CRLBs of the three models are almost equivalent. This is because the high-order components are not significant with short observation times. It can be seen that the CRLB of the TF models is inversely proportional to the observation time *T*. When $T\;>\;T_{\gamma_{1}}$, as the TFRF and TVD models provide more information for localization, increasing the observation time results in better localization accuracy. Besides, the CRLB of the TVD model is almost the same as that of TFRF model. This is because the higher order of time delay, such as the second-order of the Doppler shift, does not provide information for the localization. Although it has no effect on localization performance, higher order components should be taken into account. It is because, for long observation times, the neglect of high-order components will lead to deterioration of the peak shape for parameter estimation or the shape of the cost function that corresponds to the map grid, which will affect the localization accuracy.

By analyzing the cost functions in Figure 3 and Figure 4 and the dotted line with the green circle mark in Figure 6, we can see that the peak shape of the TF model becomes broader when T≥0.1 s, and the DPD-TF algorithm cannot reach the CRLB. Furthermore, as the observation time *T* increases, the localization accuracy gradually deteriorates. For the 2-step TF algorithm labeled with a dotted line with the purple diamond shape, similar to the results in [32], the FDOA estimation accuracy decreases as the observation time increases for T≥0.1 s, which also leads to a gradual deterioration in the localization accuracy. When the observation time is $T<T_{\gamma_{2}}$, the accuracy of the 2-step TFRF algorithm can achieve a corresponding CRLB. However, when $T>T_{\gamma_{2}}$, it can not achieve the CRLB, and the performance becomes worse as *T* increases. As shown by the dotted line with the red cross mark, the algorithm of the proposed TVD model can achieve the CRLB under the given observation times.

### 5.4. Dependence on SNR

**Figure 7:**
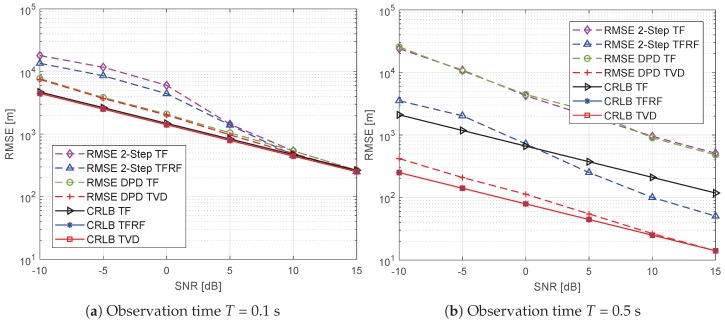
RMSE and CRLB versus SNR.

Figure 7 shows the localization performance versus SNR for observation times of *T* = 0.1 and 0.5 s. The SNR ranges from −10 to 15 dB, and the signal bandwidth is *B* = 40 kHz. As depicted in Figure 7a, when *T* = 0.1 s, the CRLBs of the three signal models are almost the same, which is similar to the conclusion we drew from Figure 6. Therefore, the 2-step TF and 2-step TFRF algorithms offer similar localization performance, and DPD-TF achieves a similar performance to the DPD-TVD algorithm. Comparing the 2-step and DPD algorithms, similar localization performance can be attained in the case of high SNRs. However, it is worth noting that the DPD algorithms can achieve better performance at low SNRs. The 2-step algorithms encounter a threshold effect when the SNR ≤ 0 dB, and the localization accuracy deteriorates sharply.

As shown in Figure 7b, when *T* = 0.5 s, the CRLBs of the TVD and TFRF models are better than that of the TF model. This is because the proposed TVD model considers higher-order components of time delay, and the new model provides more information under highly dynamic scenarios with long observation times. Note that in the two figures, the CRLB curves of different models with different observation times are parallel, which indicates that SNR has the same influence on these algorithms. As for the RMSEs of algorithms, the 2-Step TF and the DPD-TF algorithm are invalid, and the localization accuracy is far from corresponding to CRLB. As *T* is greater than $T_{\gamma_{2}}$, the 2-step TFRF cannot be close to the CRLB either. The DPD-TVD approaches the CRLB at high SNRs, but cannot when SNR ≤ 5 dB.

### 5.5. Dependence on the Signal Bandwidth *B*

**Figure 8:**
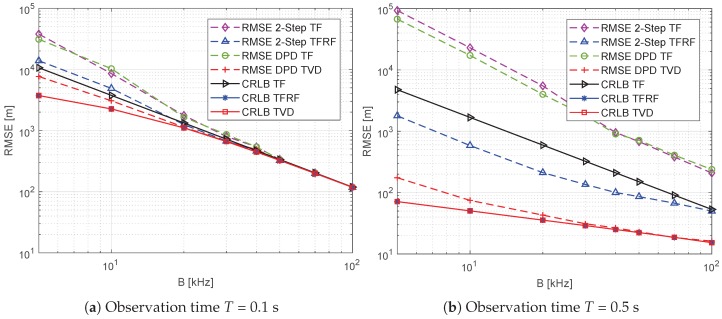
RMSE and CRLB versus *B*.

Figure 8 shows the localization performance versus the signal bandwidth *B* for observation signal times of *T* = 0.1 and 0.5 s. The signal bandwidth *B* ranges from 5 to 100 kHz and the SNR is 10 dB. According to the theoretical accuracy of TDOA and FDOA in [31], the accuracy of TDOA is mainly determined by the bandwidth *B*, while that of FDOA is determined by the observation time *T*.

In Figure 8a, the observation time is *T* = 0.1 s. When B≥ 40 kHz, the CRLBs of the three signal models are almost the same. When B≤ 40 kHz, the CRLBs of TFRF and TVD models are better than that of TF model. The 2-step TF and 2-step TFRF algorithms offer similar localization performance, and DPD-TF achieves similar performance to the DPD-TVD algorithm. The RMSEs of algorithms can get close to the corresponding CRLBs when B> 20 kHz. The accuracy of algorithms with small *B* values is affected by the threshold effect like what happens at low SNRs. Compared with the two-step algorithms, the DPD algorithms can achieve better accuracy when B≤ 20 kHz.

In Figure 8b, when *T* = 0.5 s, the CRLB of TF model is significantly different from that of TVD model. This is because high-order components can provide more information for localization with long observation times. The CRLB curve of the TF model is not parallel to that of the TVD model. The slope of the CRLB TVD curve is smaller, that is, the localization accuracy changes less with the change of *B*, which means that *B* has less influence on the localization in this case. By comparing the two figures, it can be found that the curves of CRLB with three signal models when *T* = 0.1 s and that of the TF model when *T* = 0.5 s are parallel. However, the curve of CRLB TVD is not. The signal bandwidth *B* has a different influence on these algorithms under different conditions, which is different from the conclusion in Figure 7. The performance of the algorithms is similar to the results in Figure 6. Under the condition of *T* = 0.5 s, the 2-step TF, 2-step TFRF, and DPD-TF algorithms are invalid. The accuracy of the DPD-TVD algorithm can be similar to the CRLB, but cannot attain the CRLB when B≤ 20 kHz.

## 6. Discussion

In this paper, the proposed time-varying delay-based DPD algorithm was used to calculate the time delay for each sampling point. Compared with the traditional methods, it requires a huge amount of computation, which is closely related to the sampling rate and the density of the geographical grid.

There are several ways to reduce the amount of computation, as follows. First of all, it is not necessary to calculate the time delay of each sampling point to get the time-varying delay. We can use a short interval with the same time delay. An appropriate length for the short interval can be determined according to the movement characteristics of the sensors. Note that the length must be short enough to reflect the change in time delay over time. Secondly, the density of the grid is closely related to the localization accuracy. The combination of coarse grid and fine grid can be considered to realize the balance between the number of grid points and the localization accuracy. In addition, according to the peak shape of the cost function, the two- or three-dimensional grid search can be transformed into a one-dimensional search by a certain path, which will greatly reduce the number of grid points to be calculated. Finally, calculating the time delay between the position of the sensor and the grid point involves a large number of operations with the same calculation mode. In practical applications, hardware, such as field–programmable gate array (FPGA) or graphics processing unit (GPU), can be used for parallel computing, and the computing capacity is becoming more and more powerful. Some repetitive steps of the algorithm can run on hardware, which can greatly improve the efficiency. We have implemented geographical grid based localization algorithm on the FPGA platform. Compared with computer-based implementation, it can greatly reduce the computing time, which will be investigated in future work.

## 7. Conclusions

In this paper, we have proposed a novel DPD algorithm based on the time-varying delay that can improve the localization accuracy, especially in highly dynamic scenarios. The proposed algorithm uses a new signal model in which the propagation time delay is regarded as time-varying. An ML cost function transforms the problem into that of finding the largest eigenvalue of a matrix. Performance comparisons against other one-step or two-step algorithms and CRLBs were conducted through a series of simulations, demonstrating the improvement in localization performance that can be achieved using the proposed DPD-TVD algorithm. 

## Abbreviations

The following abbreviations are used in this manuscript:

TVD	time-varying delay
DPD	direct position determination
AOA	angle of arrival
RSS	received signal strength
TOA	time of arrival
TDOA	time difference of arrival
FDOA	frequency difference of arrival
RFDOA	rate of frequency difference of arrival
ML	maximum-likelihood
CAF	complex ambiguity function
CRLB	Cramer–Rao lower bound
RTC	relative time companding
RDC	relative Doppler companding
BPSK	binary phase shift keying
SNR	signal-to-noise ratio
RMSE	root mean squared error
FPGA	field–programmable gate array
GPU	graphics processing unit
